# Achieving inactive disease state in men and women with axial spondyloarthritis: results from a multi-country prospective observational study

**DOI:** 10.1093/rheumatology/keaf447

**Published:** 2025-08-20

**Authors:** Denis Poddubnyy, Joachim Sieper, Servet Akar, Santiago Muñoz-Fernández, Hildrun Haibel, Mikhail Protopopov, Elisabeth Altmaier, Fabiana Ganz, Robert D Inman

**Affiliations:** Division of Rheumatology, University of Toronto, Toronto, ON, Canada; Schroeder Arthritis Institute, University Health Network, University of Toronto, Toronto, ON, Canada; Department of Gastroenterology, Infectious Diseases and Rheumatology, Charité-Universitätsmedizin Berlin, Berlin, Germany; Department of Gastroenterology, Infectious Diseases and Rheumatology, Charité-Universitätsmedizin Berlin, Berlin, Germany; Division of Rheumatology, Department of Internal Medicine, Izmir Katip Celebi University, Izmir, Turkey; Servicio de Reumatología, Hospital Universitario Infanta Sofía, Universidad Europea, Madrid, Spain; Department of Gastroenterology, Infectious Diseases and Rheumatology, Charité-Universitätsmedizin Berlin, Berlin, Germany; Department of Gastroenterology, Infectious Diseases and Rheumatology, Charité-Universitätsmedizin Berlin, Berlin, Germany; GKM Gesellschaft für Therapieforschung mbH, Munich, Germany; Global Medical Affairs, AbbVie Inc., Baar, Switzerland; Division of Rheumatology, University of Toronto, Toronto, ON, Canada; Schroeder Arthritis Institute, University Health Network, University of Toronto, Toronto, ON, Canada

**Keywords:** axial spondyloarthritis, sex, TNF-blocking antibody, real-world data

## Abstract

**Objective:**

To assess the probability of achieving low disease activity (LDA) and inactive disease (ID) in men *vs* women with recently diagnosed axial spondyloarthritis (axSpA).

**Methods:**

This 1-year post-hoc analysis of the PROOF study assessed patients with recently diagnosed (≤12 months) axSpA. Patients were classified as having radiographic (r) or non-radiographic (nr) axSpA using central radiograph readings. Demographics, disease characteristics, and treatment were assessed at baseline and annually thereafter. Disease activity at 1 year was assessed by Axial Spondyloarthritis Disease Activity Score (ASDAS).

**Results:**

1385 patients with baseline and year 1 data were included. Patients were classified as having nr-axSpA (*n* = 477 [34%]; 47% men) or r-axSpA (*n* = 908 [66%]; 71% men). Men had higher TNF inhibitor (TNFi) use *vs* women with nr-axSpA (15% *vs* 9%; *P = *0.0238) and r-axSpA (20% *vs* 13%; *P = *0.0118). At 1 year, TNFi use increased, remaining significantly higher for men with r-axSpA (34.9% *vs* 28.1%; *P = *0.0497) but not nr-axSpA. Significantly more men with nr-axSpA achieved ASDAS LDA (46.0% *vs* 27.1%; *P = *0.0002) and ID (21.7% *vs* 9.6%; *P = *0.0013); no significant difference was observed in r-axSpA. Female sex was associated with significantly lower odds of achieving LDA and ID in nr-axSpA only (odds ratio, men *vs* women, LDA: 2.23 [95% CI, 1.45, 3.42]; ID: 2.40 [1.39, 4.12]).

**Conclusion:**

Women with nr-axSpA, but not r-axSpA, were less likely to achieve LDA and ID after 1 year than men. These data stress the need to carefully evaluate the diagnosis and causes of pain in patients diagnosed with axSpA.

Rheumatology key messages

- Women with nr-axSpA but not r-axSpA were significantly less likely to achieve low disease activity and inactive disease compared with men.
- This effect was observed among women versus men with nr-axSpA treated with or without tumour necrosis factor inhibitor.
- A careful evaluation of the source of symptoms is necessary in the case of treatment non-response, especially in women diagnosed with nr-axSpA.

## Introduction

Axial spondyloarthritis (axSpA), which includes radiographic axSpA (r-axSpA) and non-radiographic axSpA (nr-axSpA), is an immune-mediated inflammatory disease primarily affecting the axial skeleton [1]. Clinical presentation of axSpA is characterized by inflammatory and chronic back pain, with peripheral manifestations, such as arthritis and enthesitis, and extra-musculoskeletal manifestations, including uveitis [1]. The global prevalence is estimated to range from 0.13% to 1.4% for axSpA and 0.01% to 0.54% for r-axSpA [2, 3].

Current epidemiological data indicate the prevalence of r-axSpA to be predominantly among male patients, with a near equal prevalence of nr-axSpA between men and women [3, 4]. Furthermore, emerging evidence from randomized, observational and registry studies suggests that men and women experience axSpA differently [4–6]. Women with axSpA tend to have a higher disease burden than men, possibly due to longer diagnostic delay, higher clinical disease activity scores and a lower treatment response [4–6]. Furthermore, on a group level, women have lower CRP levels, develop less structural damage in the sacroiliac joints and spine, and have slower radiographic progression than men [7–9].

The PROOF study was a global, observational study in patients with recently diagnosed (≤12 months) axSpA in real-world rheumatology clinical practices [10]. The baseline results of PROOF demonstrated significant differences in the proportion of men *vs* women classified as having nr-axSpA (48.5% *vs* 51.5%, respectively) and r-axSpA (71.0% *vs* 29.0%) [10].

The main objective of this post-hoc analysis of the PROOF study is to assess the probability of achieving low disease activity and inactive disease after 1 year of follow-up, in the context of demographic and clinical characteristics, as well as treatment, in men *vs* women with recently diagnosed nr-axSpA or r-axSpA.

## Methods

PROOF was a 5-year, real-world, prospective, observational study conducted in rheumatology clinical practices in 29 countries across six geographic regions in patients recently diagnosed with axSpA by rheumatologists. The methods and baseline results of PROOF have been reported previously [10]. Briefly, the study enrolled adult patients with recently diagnosed (≤12 months) axSpA who also fulfilled the Assessment of Spondyloarthritis International Society (ASAS) classification criteria. Patients were further classified as having either r-axSpA or nr-axSpA based on central evaluation of sacroiliac radiographs; grading was in accordance with the modified New York criteria for axial spondyloarthritis [11, 12]. Patients with sacroiliitis of grade ≥2 bilaterally or grade ≥3 unilaterally were classified as having r-axSpA; otherwise, they were classified as having nr-axSpA. The radiographs were assessed first by a local reader, followed by a central reader. In case of a disagreement in the classification (r- *vs* nr-axSpA) between the local and central reader, the radiograph was evaluated by a second central reader (adjudicator) who was blinded to the previous assessments, and this assessment determined the final classification. For baseline data, the local classification was retained for 83% of patients after central reading [10].

The study was approved by local ethics committees of each study site in accordance with local laws and regulations and conducted in accordance with the Declaration of Helsinki. Patients provided written informed consent and written authorization to send anonymized imaging of sacroiliac joints to a central reading centre [10].

Demographics, clinical disease characteristics and treatments were assessed at baseline and annually thereafter. Information on sex (female or male) was collected at baseline; no information on gender beyond sex was obtained. Patients included in the analysis had both baseline and year 1 data available.

Disease activity at 1 year was assessed by the proportion of patients achieving Axial Spondyloarthritis Disease Activity Score (ASDAS) inactive disease (ID; defined as ASDAS <1.3), and ASDAS low disease activity (LDA; defined as ASDAS <2.1). In addition, we evaluated the following outcomes: ASDAS clinically important improvement (CII; defined as improvement ≥1.1 compared with baseline), ASDAS major improvement (MI; defined as improvement ≥2.0 compared with baseline), and Bath Ankylosing Spondylitis Disease Activity Index (BASDAI) ≤4 (*vs* >4).

### Statistical analyses

In the analysis comparing crude proportions of patients achieving ID and LDA, patients were stratified according to r-axSpA or nr-axSpA classification and sex. Univariable regression analysis was conducted in r- and nr-axSpA subgroups. Multivariable regression analyses of factors associated with ASDAS LDA or ASDAS ID in nr-axSpA and r-axSpA populations included sex, TNF inhibitor (TNFi) use at 1 year, symptom duration (months), HLA-B27 positivity, age (years), CRP level at baseline (mg/l), BASDAI score at baseline, and presence of fibromyalgia as independent variables. Each analysis was additionally conducted in nr-axSpA and r-axSpA populations with and without TNFi use. The selection of the independent variables was based on the authors’ knowledge of the factors potentially associated with treatment response. Descriptive analyses comparing baseline and year 1 characteristics of men *vs* women were conducted using the Mann–Whitney test and χ^2^ test, as appropriate for the variable type. *P*-values <0.05 were considered as indicators of statistical significance. No multiple testing correction was performed. All data were reported as observed; no imputation of missing data was performed. All statistical analyses were performed using the SAS package (version 9.4; SAS Institute, Cary, NC, USA).

## Results

Of 2633 enrolled patients, 1612 underwent central reading of radiographs; 617 patients were excluded as pelvic radiographs were not provided to the central reading centre and standardized classification was not possible. Of patients with central reading, 1385 had baseline and year 1 data and were classified as having nr-axSpA (477 [34%]) or r-axSpA (908 [66%]) at baseline (Supplementary Fig. S1). Baseline characteristics for patients without central reading who also did not have both baseline and 1-year data and were therefore excluded from the analyses are provided in Supplementary Table S1. Overall, the disease characteristics appeared to be comparable, except for the presence of MRI inflammation, which is suggestive of SpA. This difference may be related to differences in clinical practice in some centres—if the MRI was performed first and revealed changes suggestive of SpA, radiographs might have been skipped and were, therefore, not available for central evaluation. Among patients with nr-axSpA, approximately half were men (*n* = 226 [47%]), whereas among patients with r-axSpA, the majority were men (*n* = 645 [71%)]; Table 1). Men were younger than women (mean age, 34.1 *vs* 37.1 years; *P = *0.0004) in the nr-axSpA population whereas no significant difference in age was observed in the r-axSpA population (34.2 *vs* 35.4 years; Table 1). Men were more frequently HLA-B27 positive and had higher levels of CRP than women in both populations. However, more women *vs* men with r-axSpA presented with active inflammation on MRI at baseline, (25.5% *vs* 11.3%), whereas no significant difference between sexes was observed in the nr-axSpA population (Table 1). Baseline disease activity measured with BASDAI was higher among women in both the nr-axSpA and r-axSpA populations (Table 1). Among patients with nr-axSpA, fibromyalgia was reported in 10 (4.0%) women and one (0.4%) man at baseline; fibromyalgia was reported in four (1.6%) additional women at 1 year.

**Table 1.** Patient characteristics at baseline stratified by sex and axSpA classification criteria

Characteristic	nr-axSpA			r-axSpA		
	Men	Women	***P*-value** ^a^	Men	Women	***P*-value** ^a^
	(*n* = 226)	(*n* = 251)		(*n* = 645)	(*n* = 263)	
Age, mean (s.d.), years	34.1 (9.8)	37.1 (9.6)	**0.0004**	34.2 (11.2)	35.4 (10.3)	0.0460
Symptom duration, mean (s.d.), months	48.6 (67.3)	53.7 (72.0)	0.4203	60.0 (88.7)	67.0 (90.8)	0.7455
Time from diagnosis to baseline visit, mean (s.d.), months	3.1 (3.2) (*n* = 164)	2.4 (3.1) (*n* = 174)	**0.0257**	3.0 (3.3) (*n* = 525)	3.0 (3.3) (*n* = 200)	0.7712
Number of SpA features^b^, mean (s.d.)	3.8 (1.5) (*n* = 199)	3.4 (1.3) (*n* = 211)	**0.0090**	4.0 (1.4) (*n* = 526)	3.5 (1.3) (*n* = 224)	<**0.0001**
SpA features, *n* (%)						
HLA-B27 positive^c^	127 (63.8) (*n* = 199)	107 (50.7) (*n* = 211)	**0.0074**	399 (75.9) (*n* = 526)	125 (55.8) (*n* = 224)	<**0.0001**
Inflammatory back pain	216 (95.6)	240 (95.6)	0.9821	617 (95.7)	251 (95.4)	0.8826
Peripheral arthritis	79 (35.0)	77 (30.7)	0.3200	216 (33.5)	88 (33.5)	0.9935
Enthesitis, heel	91 (40.3)	90 (35.9)	0.3218	219 (34.0)	88 (33.5)	0.8866
Dactylitis	14 (6.2)	12 (4.8)	0.4970	35 (5.4)	12 (4.6)	0.5942
Uveitis	25 (11.1)	22 (8.8)	0.4006	76 (11.8)	22 (8.4)	0.1322
Psoriasis	32 (14.2)	24 (9.6)	0.1193	37 (5.7)	11 (4.2)	0.3425
IBD	8 (3.5)	10 (4.0)	0.7993	10 (1.6)	6 (2.3)	0.4476
Good response to NSAIDs	144 (63.7)	142 (56.6)	0.1119	387 (60.0)	167 (63.5)	0.3269
Family history of SpA	43 (19.0)	49 (19.5)	0.8911	124 (19.2)	43 (16.3)	0.3104
Elevated CRP	85 (37.6)	74 (29.5)	0.0601	383 (59.4)	99 (37.6)	**<0.0001**
CRP, mean (s.d.), mg/l	14.1 (22.2) (*n* = 201)	9.9 (17.3) (*n* = 218)	**0.0121**	18.8 (24.8) (*n* = 563)	13.1 (21.4) (*n* = 228)	**<0.0001**
ASDAS-CRP, mean (s.d.)	2.8 (1.2)	2.8 (1.1)	0.9237	3.0 (1.1)	2.9 (1.1)	0.3692
BASDAI, mean (s.d.)	4.4 (2.4)	5.0 (2.4)	**0.0036**	4.2 (2.2)	4.7 (2.3)	**0.0015**
BASFI, mean (s.d.)	3.1 (2.5)	3.6 (2.4)	**0.0388**	3.3 (2.5)	3.7 (2.6)	**0.0329**
Active inflammation on MRI highly suggestive of sacroiliitis associated with SpA^d^, *n* (%)	107 (47.3)	132 (52.6)	0.5724	73 (11.3)	67 (25.5)	**<0.0001**

a For the comparison of male *vs* female sex using Mann–Whitney and χ^2^ tests.

b SpA features included in the ASAS classification criteria for axSpA, excluding imaging.

c Based on patients with HLA-B27 assessed.

d As assessed by the investigator; the images could have been performed in the past. Bold text indicates significant *P*-values. ASAS: Assessment of Spondyloarthritis International Society; ASDAS-CRP: Axial Spondyloarthritis Disease Activity Score containing CRP; axSpA: axial spondyloarthritis; nr-axSpA: non-radiographic axial spondyloarthritis; NSAID: non-steroidal anti-inflammatory drug; r-axSpA: radiographic axial spondyloarthritis; SpA: spondyloarthritis.

Interestingly, men had significantly higher TNFi use in both the nr-axSpA (15% *vs* 9%; *P = *0.0238) and r-axSpA (20% *vs* 13%; *P = *0.0118) populations at baseline (Table 2). At 1 year, TNFi use had increased overall and remained significantly higher for men *vs* women with r-axSpA but not with nr-axSpA. Women with r-axSpA had higher use of analgesics *vs* men at baseline and at 1 year, but no significant difference between the sexes in analgesic use was observed among patients with nr-axSpA (Table 2). Non-steroidal anti-inflammatory drug use was higher among women *vs* men with nr-axSpA at 1 year.

**Table 2.** Baseline and year 1 treatments stratified by sex and axSpA classification criteria

Treatment, *n* (%)	nr-axSpA			r-axSpA		
	Men	Women	***P*-value** ^a^	Men	Women	***P*-value** ^a^
	(*n* = 226)	(*n* = 251)		(*n* = 645)	(*n* = 263)	
Baseline						
NSAIDs	175 (77.4)	197 (78.5)	0.7818	496 (76.9)	211 (80.2)	0.2731
csDMARDs	62 (27.4)	73 (29.1)	0.6896	207 (32.1)	84 (31.9)	0.9641
Analgesics	38 (16.8)	46 (18.3)	0.6650	81 (12.6)	48 (18.3)	**0.0258**
TNFi	35 (15.5)	22 (8.8)	**0.0238**	129 (20.0)	34 (12.9)	**0.0118**
Year 1						
NSAIDs	132 (58.4)	182 (72.5)	**0.0012**	437 (67.8)	179 (68.1)	0.9280
csDMARDs	59 (26.1)	63 (25.1)	0.8014	204 (31.6)	77 (29.3)	0.4871
Analgesics	31 (13.7)	33 (13.1)	0.8554	60 (9.3)	38 (14.4)	**0.0234**
TNFi	67 (29.6)	66 (26.3)	0.4151	225 (34.9)	74 (28.1)	**0.0497**

a For the comparison of male *vs* female sex using χ^2^ tests. Bold text indicates significant *P*-values. axSpA: axial spondyloarthritis; csDMARD: conventional systemic DMARD; nr-axSpA: non-radiographic axial spondyloarthritis; NSAID: non-steroidal anti-inflammatory drug; r-axSpA: radiographic axial spondyloarthritis; TNFi: TNF inhibitor.

**Figure 1.**
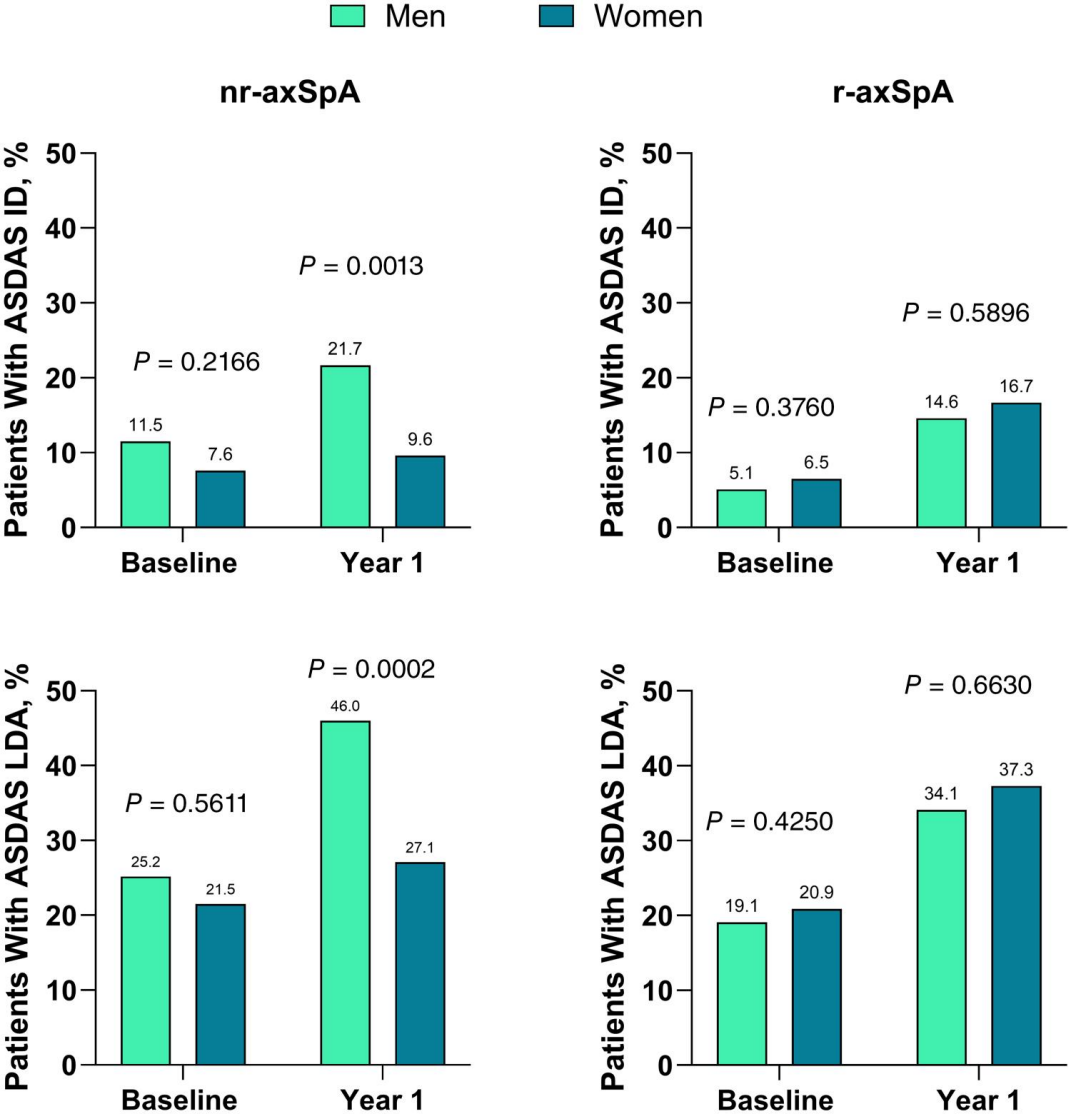
Proportion of patients with ASDAS ID and ASDAS LDA at baseline and year 1 among men and women across nr-axSpA and r-axSpA populations. ASDAS: Axial Spondyloarthritis Disease Activity Score; ID: inactive disease (ASDAS <1.3); LDA: low disease activity (ASDAS <2.1); nr-axSpA: non-radiographic axial spondyloarthritis; r-axSpA: radiographic axial spondyloarthritis

**Figure 2.**
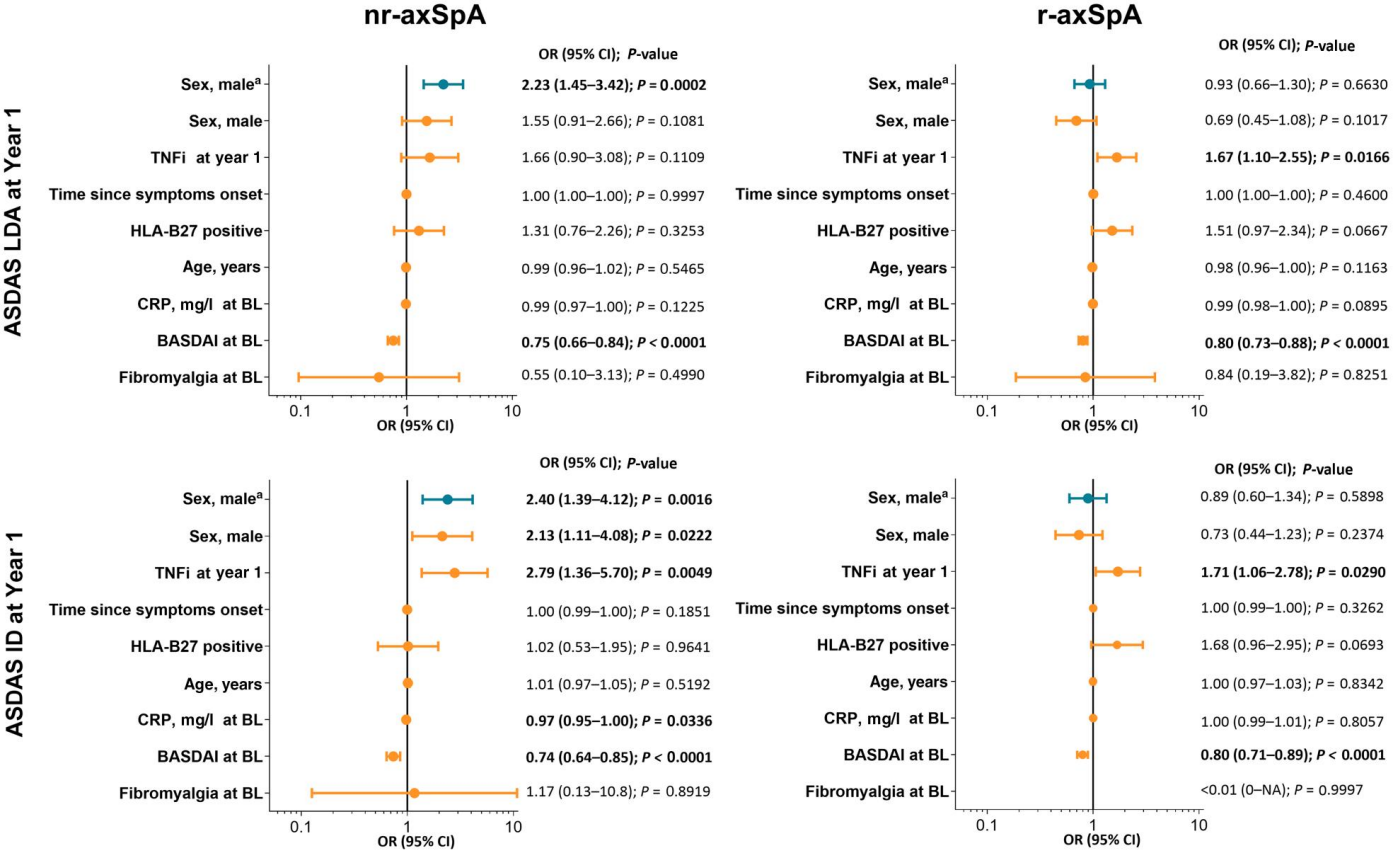
Regression analysis of factors associated with ASDAS LDA and ASDAS ID in nr-axSpA and r-axSpA populations at year 1. ^a^Results from a univariable regression analysis. ASDAS: Axial Spondyloarthritis Disease Activity Score; BL: baseline; ID: inactive disease (ASDAS <1.3); LDA: low disease activity (ASDAS <2.1); nr-axSpA: non-radiographic axial spondyloarthritis; OR: odds ratio; r-axSpA: radiographic axial spondyloarthritis; TNFi: TNF inhibitor; NA: not applicable

Disease activity generally improved from baseline to 1 year for men and women in both populations (Fig. 1). However, significantly more men *vs* women in the nr-axSpA population achieved ASDAS ID (21.7% *vs* 9.6%, respectively) and ASDAS LDA (46.0% *vs* 27.1%, respectively) at 1 year, but there were no significant differences in the r-axSpA group (ASDAS ID, 14.6% *vs* 16.7%, respectively; ASDAS LDA, 34.1% *vs* 37.3%, respectively). Female sex was associated with significantly lower odds of achieving ASDAS LDA (men *vs* women: odds ratio [OR], 2.23 [95% CI, 1.45, 3.42]) and ID (2.40 [1.39, 4.12]) in patients with nr-axSpA but not with r-axSpA (men *vs* women, LDA: 0.93 [0.66, 1.30]; ID: 0.89 [0.60, 1.34]). The association with ASDAS ID was present also in the multivariable models including TNFi use at 1 year, symptom duration, HLA-B27 positivity, sex, age, CRP level at baseline, BASDAI score at baseline and presence of fibromyalgia (Fig. 2).

Women without TNFi use had significantly higher disease activity (assessed with BASDAI) *vs* men at baseline in both the nr-axSpA (Supplementary Table S2) and r-axSpA (Supplementary Table S3) populations. Furthermore, significantly more women *vs* men with r-axSpA had active sacroiliitis identified by MRI (local reading) at baseline regardless of TNFi use (Supplementary Table S3). In contrast, significantly more men *vs* women with r-axSpA had elevated CRP levels, regardless of TNFi use (TNFi users, 66.7% *vs* 48.6% [*P = *0.0056]; no TNFi use, 55.5% *vs* 33.3% [*P *< 0.001]), and occurrences of uveitis (15.1% *vs* 4.1%; *P = *0.0122) in TNFi users but not among non-users (Supplementary Table S3).

**Figure 3.**
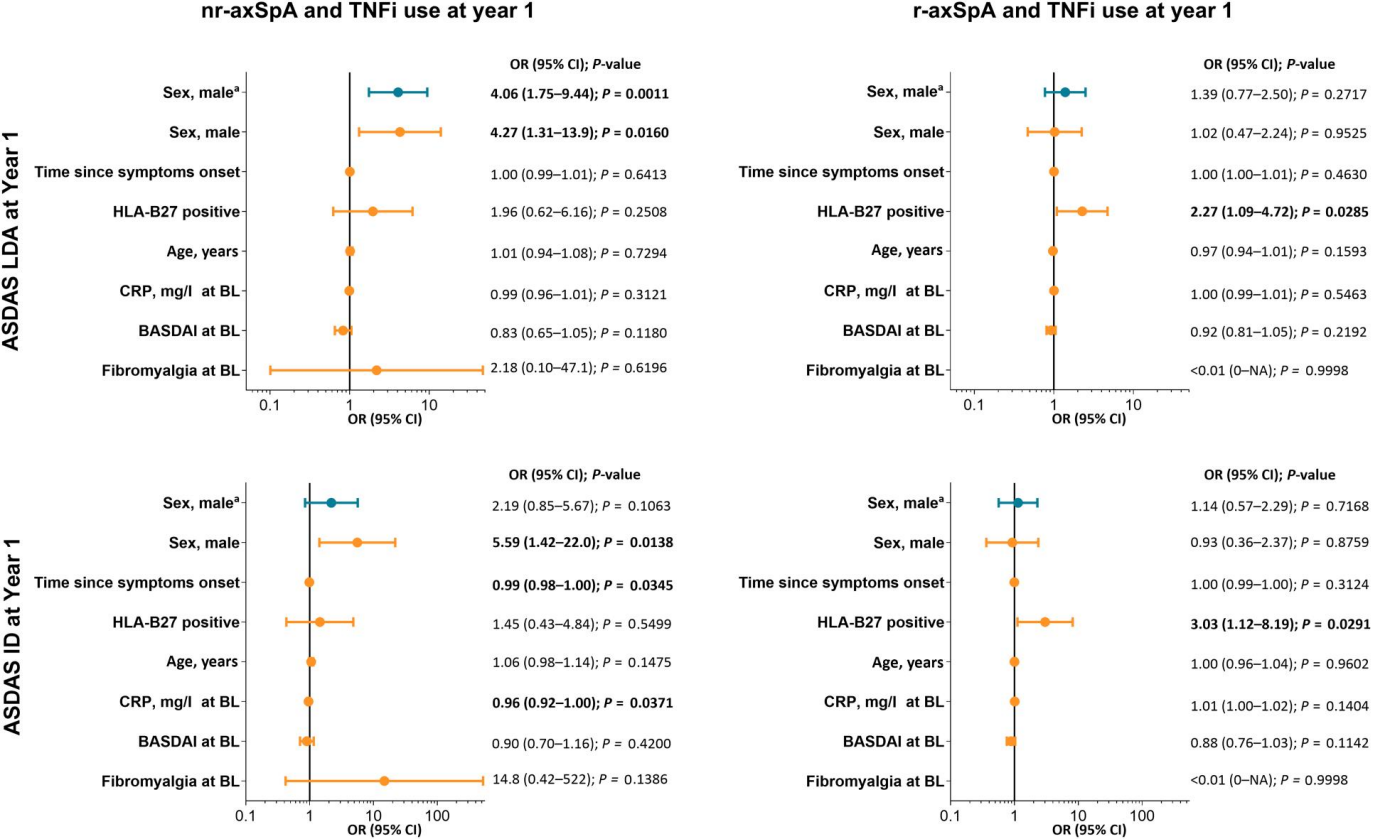
Regression analysis of factors associated with ASDAS LDA and ASDAS ID in nr-axSpA and r-axSpA populations with TNFi use at year 1. ^a^Results from a univariable regression analysis. ASDAS: Axial Spondyloarthritis Disease Activity Score; BL: baseline; ID: inactive disease (ASDAS <1.3); LDA: low disease activity (ASDAS <2.1); nr-axSpA: non-radiographic axial spondyloarthritis; OR: odds ratio; r-axSpA: radiographic axial spondyloarthritis; TNFi: TNF inhibitor; NA: not applicable

**Figure 4.**
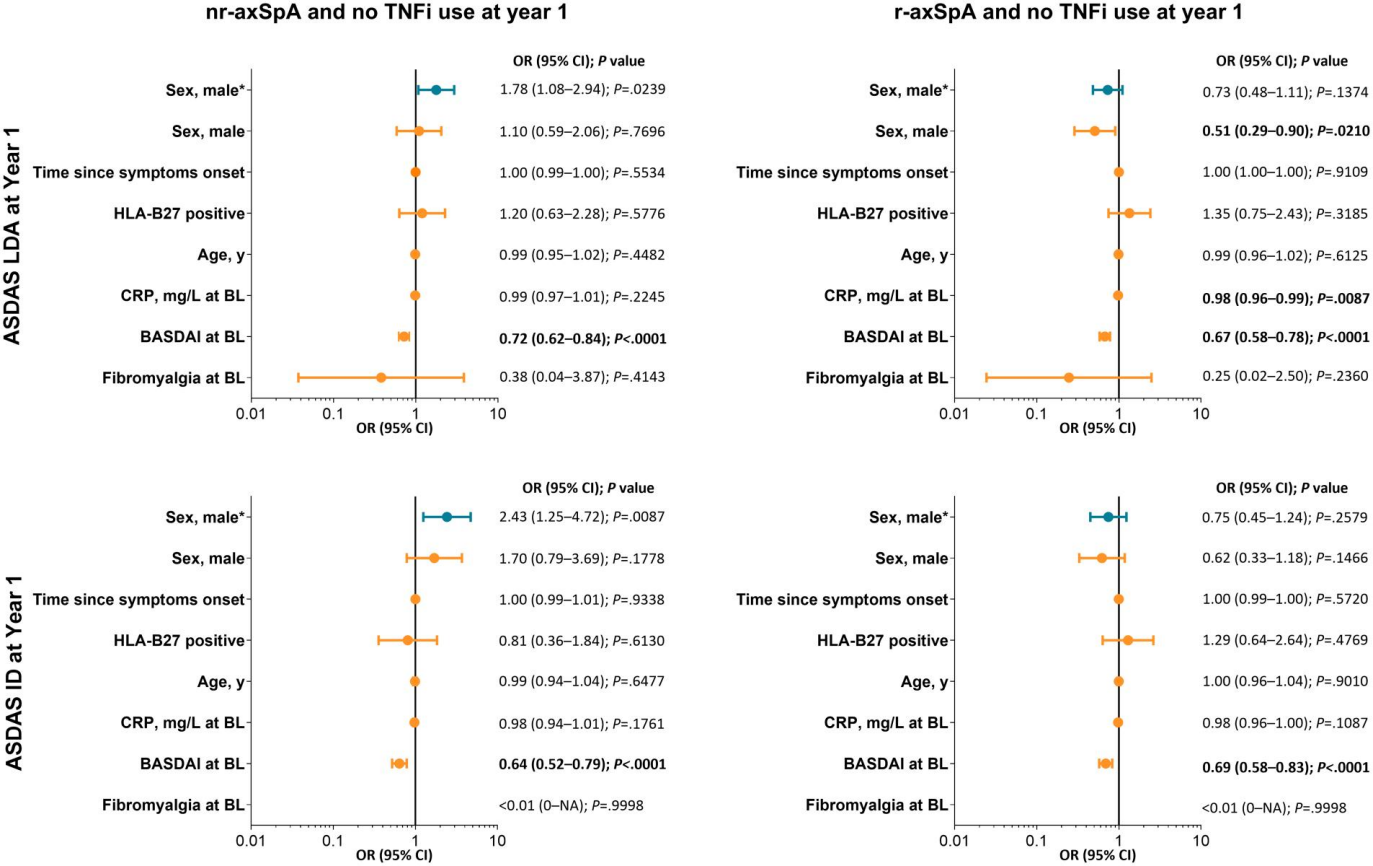
Regression analysis of factors associated with ASDAS LDA and ASDAS ID in nr-axSpA and r-axSpA populations without TNFi use at year 1. ^a^Results from a univariable regression analysis. ASDAS: Axial Spondyloarthritis Disease Activity Score; BL: baseline; ID: inactive disease (ASDAS <1.3); LDA: low disease activity (ASDAS <2.1); nr-axSpA: non-radiographic axial spondyloarthritis; OR: odds ratio; r-axSpA: radiographic axial spondyloarthritis; TNFi: TNF inhibitor; NA: not applicable

Among patients with TNFi use at 1 year, female sex was associated with significantly lower odds of achieving ASDAS LDA (men *vs* women: OR 4.06 [95% CI, 1.75, 9.44] in the univariable analysis; OR 4.27 [1.31, 13.9] in the multivariable model) and ASDAS ID (men *vs* women: OR 2.19 [0.85, 5.67] in the univariable analysis and OR 5.59 [1.42, 22.0] in the multivariable model) in patients with nr-axSpA but not with r-axSpA (Fig. 3). Among patients with nr-axSpA who had not used a TNFi at 1 year, female sex was associated with significantly lower odds of achieving ASDAS LDA or ASDAS ID (men *vs* women, LDA: 1.78 [95% CI, 1.08, 2.94]; ID: 2.43 [1.25, 4.72]) in the univariable analysis but not in the multivariable model (Fig. 4).

## Discussion

In this study, women with nr-axSpA, but not r-axSpA, were significantly less likely to achieve ASDAS ID and LDA at year 1 compared with men. Likelihood of achieving ASDAS ID and LDA was also lower among women *vs* men with nr-axSpA who used a TNFi at 1 year, suggesting that non-inflammatory mechanisms might contribute to the presence of pain and symptoms in women diagnosed with nr-axSpA including those receiving TNFi treatment. One of the possible explanations might be non-nociceptive (e.g. nociplastic) pain mechanisms or related to mechanical problems in the sacroiliac joints responsible for bone marrow oedema visible on MRI (such as osteitis condensans ilii, which primarily affects women) [13, 14]. This might also be associated with a lack of response to NSAIDs and the escalation to TNFi, which in turn resulted in a somewhat stronger association between sex and achieving good treatment response in TNFi-treated patients. In r-axSpA, the presence of (centrally confirmed) definite radiographic sacroiliitis indicates an inflammatory origin of the disease and its symptoms, and might, therefore, be associated with a lower risk of misdiagnosis.

In our view, that may relate to the results showing no difference between men and women with r-axSpA in terms of response achievement. A number of studies have reported a worse response to biologic (b)DMARDs in female *vs* male patients with axSpA (including both radiographic and non-radiographic forms) [15, 16]. However, the PROOF study is the largest existing cohort to date in which classification was performed centrally and independently of clinical assessment for all patients. A recent analysis of a large cohort from Toronto, Canada showed a higher risk for treatment discontinuation in nr- *vs* r-axSpA [17]. There are no doubts that there are biological and social differences between men and women with axSpA, potentially influencing perception of symptoms, response to anti/inflammatory treatment, as well as differences in their patient journeys [18]. However, rheumatologists should also be aware of the potential for misdiagnosis in cases of non-specific bone marrow oedema, which appears to be more common in women compared with men with axSpA [19]. This may be explained by a higher prevalence of mechanically induced bone marrow oedema in women, particularly related to osteitis condensans ilii (a mechanical condition strongly associated with prior pregnancies and deliveries and showing a clear female predominance [13]), as well as by a higher frequency of anatomical variations in sacroiliac joint morphology [20, 21]. The risk of misdiagnosis appears to be higher in the absence of radiographic sacroiliitis explaining the differences between nr- and r-axSpA.

Our findings also showed that women were significantly older than men in the nr-axSpA population at baseline (although mean time from diagnosis to baseline visit was shorter for women: 2.4 *vs* 3.1 months). Higher HLA-B27 positivity, greater CRP levels, and more radiographic damage was observed among men *vs* women with axSpA, which is consistent with previous studies [4, 5]. We also showed that women tended to have higher disease activity as assessed by BASDAI indicating higher symptom burden than men in both the nr-axSpA and r-axSpA populations. Interestingly, more women *vs* men with r-axSpA presented with active sacroiliitis on MRI at baseline (according to the local assessment) regardless of TNFi use in the PROOF study. That was not the case in nr-axSpA patients. However, since MRI of sacroiliac joints was performed as a part of routine care and was evaluated locally, we did not include this variable in our analysis given a high risk of bias. Of note, no use of targeted synthetic (ts)DMARDs (e.g. Janus kinase inhibitors) or bDMARDs other than TNFi (e.g. IL-17 inhibitors) was recorded during this study. This study was conducted between 2014 and 2019, and many countries did not provide cost reimbursement for IL-17 inhibitors or tsDMARDs.

Although there were only a few cases overall, a greater proportion of women with nr-axSpA had concomitant fibromyalgia (that can be considered as an extreme phenotype of central sensitization/nociplastic pain) *vs* men at baseline, and this increased by 1 year. Fibromyalgia can be a potential contributor to a higher perception of pain in women, influencing patient-reported outcomes and disease activity scores [22, 23]. In our analysis, only a few patients had fibromyalgia, so the effect estimation for this factor is not very precise. In general, central sensitization (the amplification of neural pain signalling within central somatosensory pathways) is a part of fibromyalgia pathophysiology and may contribute to an insufficient response to anti-inflammatory treatment [24, 25].

In this study, women were significantly less likely to be HLA-B27 positive. Thus, to fulfil the ASAS classification criteria for nr-axSpA, patients had to have sacroiliitis on imaging, suggesting that healthcare providers rely on MRI (bone marrow oedema) in the diagnosis of nr-axSpA [1]. However, it has been reported in the general population that factors such as increased body mass index and the history of childbirth within 12 months before MRI examination are also associated with a higher chance of having inflammatory lesions on MRI among women, highlighting the possibility of mechanical genesis of such lesions [26]. As such, relying on the presence of bone marrow oedema in HLA-B27-negative women could lead to misdiagnosis of nr-axSpA, especially after childbirth.

Strengths of this analysis include the use of a large multinational cohort and central evaluation of radiographs, allowing for valid and reliable classifications. Furthermore, inclusion of both nr-axSpA and r-axSpA populations allowed for a side-by-side stratified analysis. Study limitations included that a considerable number of participants did not have a 1-year follow-up radiograph. MRIs of sacroiliac joints, obtained as a part of clinical routine, were not centrally evaluated, and therefore there was no possibility to validate the investigator’s judgement on the presence of MRI changes suggestive of SpA. Additionally, occurrence and timing of pregnancies and childbirth were not recorded. The use of analgesics was reported to be higher in women; however, results did not differentiate between opioids and non-opioid analgesics. Fibromyalgia was captured as a comorbidity; however, no specific instruments of evaluation of central sensitization were applied, leading to a potential underestimation of cases with non-nociceptive pain. Furthermore, data from each region may not be entirely representative of a larger axSpA population in that region because investigational sites were not selected by a standardized method. Additionally, the study was conducted between 2014 and 2019, limiting bDMARD and tsDMARD treatment options to TNFi only.

In summary, the observed differences in ID and LDA achievement between men and women with nr-axSpA, especially in those treated with TNFi, might be related not only to biological differences but also to other factors not directly related to SpA. In r-axSpA, no differences between men and women could be observed. These data stress the need for careful evaluation of the diagnosis and causes of pain in patients diagnosed with axSpA presenting without relevant structural damage in the sacroiliac joints and not responding sufficiently to anti-inflammatory treatment.

## Supplementary Material

keaf447_Supplementary_Data

## Data Availability

AbbVie is committed to responsible data sharing regarding the clinical trials they sponsor. This includes access to anonymized, individual, and trial-level data (analysis data sets), as well as other information (e.g. protocols, clinical study reports, or analysis plans), as long as the trials are not part of an ongoing or planned regulatory submission. This includes requests for clinical trial data for unlicensed products and indications. These clinical trial data can be requested by any qualified researchers who engage in rigorous, independent, scientific research, and will be provided following review and approval of a research proposal, Statistical Analysis Plan (SAP), and execution of a Data Sharing Agreement (DSA). Data requests can be submitted at any time after approval in the USA and Europe and after acceptance of this manuscript for publication. The data will be accessible for 12 months, with possible extensions considered. For more information on the process or to submit a request, visit the following link: https://vivli.org/ourmember/abbvie/ then select ‘Home’.
