# Supplementary material for: Achieving inactive disease state in men and women with axial spondyloarthritis: results from a multi-country prospective observational study
Source: Rheumatology (Oxford). 2025 Aug 20;64(12):6337–44. doi: 10.1093/rheumatology/keaf447 (PMC12671858; doi:10.1093/rheumatology/keaf447)

Supplementary Figure S1. Patient disposition. ASAS, Assessment of Spondyloarthritis International Society; axSpA, axial spondyloarthritis; nr-axSpA, non-radiographic axial spondyloarthritis; r-axSpA, radiographic axial spondyloarthritis.

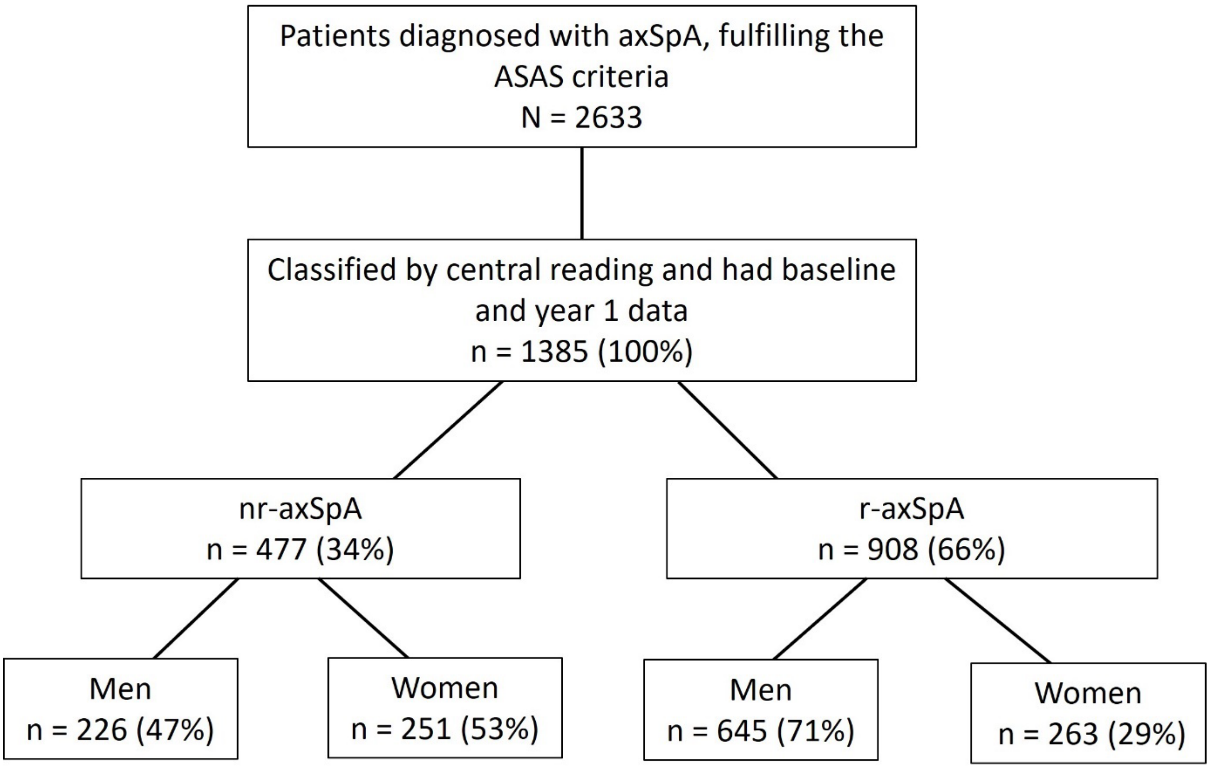

Supplement: keaf447_Supplementary_Data [file keaf447_supplementary_data.zip › keaf447_Supplementary_Data/rhe-24-1010-File006.pdf]
