# Supplementary material for: Achieving inactive disease state in men and women with axial spondyloarthritis: results from a multi-country prospective observational study
Source: Rheumatology (Oxford). 2025 Aug 20;64(12):6337–44. doi: 10.1093/rheumatology/keaf447 (PMC12671858; doi:10.1093/rheumatology/keaf447)
Supplement: keaf447_Supplementary_Data [file keaf447_supplementary_data.zip › keaf447_Supplementary_Data/rhe-24-1010-File007.docx]

**Supplementary Table S1. Patient characteristics at baseline stratified by sex and axSpA classification criteria from patients excluded from analysis without central reading and both baseline and 1-year data**

| **Characteristic** | **Included patients with central reading**  **(N = 1385)** | **Excluded patients**  **(N = 780)** |
| --- | --- | --- |
|  |  |  |
| Age, y, mean (SD) | 35.0 (10.6) | 34.0 (10.2) |
| Symptom duration, mo, mean (SD) | 58.3 (83.3) | 59.6 (81.0) |
| Time from diagnosis to baseline visit, mo, mean (SD) | 2.9 (3.3)  n = 1063 | 3.2 (3.6)  n = 614 |
| Number of SpA features,^a^ mean (SD) | 3.8 (1.4)  n = 1160 | 3.7 (1.5)  n = 657 |
| SpA features, n (%) |  |  |
| HLA-B27 positive^b^ | 758 (65.3)  n = 1160 | 449 (68.3)  n = 657 |
| Inflammatory back pain | 1324 (95.6) | 724 (92.8) |
| Peripheral arthritis | 460 (33.2) | 250 (32.1) |
| Enthesitis, heel | 488 (35.2) | 258 (33.1) |
| Dactylitis | 73 (5.3) | 52 (6.7) |
| Uveitis | 145 (10.5) | 75 (9.6) |
| Psoriasis | 104 (7.5) | 58 (7.4) |
| IBD | 34 (2.5) | 25 (3.2) |
| Good response to NSAIDs | 840 (60.6) | 420 (53.8) |
| Family history of SpA | 259 (18.7) | 152 (19.5) |
| Elevated CRP | 641 (46.3) | 328 (42.1) |
| CRP, mg/L, mean (SD) | 15.4 (22.8)  n = 1210 | 15.6 (23.5)  n = 633 |
| ASDAS-CRP, mean (SD) | 2.9 (1.1) | 2.9 (1.1) |
| BASDAI, mean (SD) | 4.5 (2.3) | 4.4 (2.3) |
| BASFI, mean (SD) | 3.4 (2.5) | 3.1 (2.5) |
| Active inflammation on MRI highly suggestive of sacroiliitis associated with SpA,^c^ n (%) | 379 (27.4) | 317 (40.6) |

ASAS, Assessment of Spondyloarthritis International Society; ASDAS-CRP, Axial Spondyloarthritis Disease Activity Score containing CRP; axSpA, axial spondyloarthritis; nr-axSpA, non-radiographic axial spondyloarthritis; NSAID, non-steroidal anti-inflammatory drug; r-axSpA, radiographic axial spondyloarthritis; SpA, spondyloarthritis.

^a^SpA features included in the ASAS classification criteria for axSpA, excluding imaging.

^b^Based on patients with HLA-B27 assessed.

^c^As assessed by the investigator; the images could have been performed in the past.
