# Supplementary material for: Achieving inactive disease state in men and women with axial spondyloarthritis: results from a multi-country prospective observational study
Source: Rheumatology (Oxford). 2025 Aug 20;64(12):6337–44. doi: 10.1093/rheumatology/keaf447 (PMC12671858; doi:10.1093/rheumatology/keaf447)
Supplement: keaf447_Supplementary_Data [file keaf447_supplementary_data.zip › keaf447_Supplementary_Data/rhe-24-1010-File008.docx]

**Supplementary Table S2. Baseline characteristics stratified by TNFi use and sex in patients with nr-axSpA**

| **Characteristic** | **TNFi use** | | | **No TNFi use** | | |
| --- | --- | --- | --- | --- | --- | --- |
|  | **Men**  **(n = 67)** | **Women**  **(n = 66)** | ***P* value^a^** | **Men**  **(n = 159)** | **Women**  **(n = 185)** | ***P* value^a^** |
| Age, y, mean (SD) | 34.6 (10.1) | 38.9 (9.9) | **.0147** | 33.9 (9.7) | 36.5 (9.4) | **.0059** |
| Symptom duration, mo, mean (SD) | 51.7 (70.4) | 46.6 (61.6) | .7753 | 47.3 (66.2) | 56.3 (75.3) | .2445 |
| Time from diagnosis to baseline visit, mo, mean (SD) | 3.8 (3.2) | 3.2 (3.2) | .2629 | 2.8 (3.1) | 2.1 (3.0) | .0818 |
| Number of SpA features,^b^ mean (SD) | 4.2 (1.4) | 3.5 (1.3) | **.0153** | 3.7 (1.5) | 3.4 (1.3) | .1425 |
| SpA features, n (%) |  |  |  |  |  |  |
| HLA-B27, positive^c^ | 37 (63.8) | 27 (54.0) | .3017 | 90 (63.8) | 80 (49.7) | **.0135** |
| Inflammatory back pain | 62 (92.5) | 65 (98.5) | .0985 | 154 (96.9) | 175 (94.6) | .3060 |
| Peripheral arthritis | 29 (43.3) | 28 (42.4) | .9202 | 50 (31.4) | 49 (26.5) | .3110 |
| Enthesitis, heel | 33 (49.3) | 21 (31.8) | **.0406** | 58 (36.5) | 69 (37.3) | .8753 |
| Dactylitis | 6 (9.0) | 1 (1.5) | .0547 | 8 (5.0) | 11 (5.9) | .7112 |
| Uveitis | 8 (11.9) | 4 (6.1) | .2367 | 17 (10.7) | 18 (9.7) | .7685 |
| Psoriasis | 18 (26.9) | 8 (12.1) | **.0320** | 14 (8.8) | 16 (8.6) | .9591 |
| IBD | 4 (6.0) | 6 (9.1) | .4950 | 4 (2.5) | 4 (2.2) | .8283 |
| Good response to NSAIDs | 38 (56.7) | 25 (37.9) | **.0296** | 106 (66.7) | 117 (63.2) | .5074 |
| Family history of SpA | 10 (14.9) | 13 (19.7) | .4669 | 33 (20.8) | 36 (19.5) | .7649 |
| Elevated CRP | 35 (52.2) | 25 (37.9) | .0961 | 50 (31.4) | 49 (26.5) | .3110 |
| CRP, mg/L, mean (SD) | 19.3 (28.7) | 11.0 (17.5) | **.0388** | 12.2 (19.0) | 9.6 (17.2) | .0882 |
| ASDAS-CRP, mean (SD) | 3.3 (1.3) | 3.0 (1.1) | .2185 | 2.6 (1.1) | 2.7 (1.1) | .4043 |
| BASDAI, mean (SD) | 5.2 (2.3) | 5.6 (2.4) | .2983 | 4.0 (2.3) | 4.8 (2.4) | **.0023** |
| BASFI, mean (SD) | 4.1 (2.6) | 4.0 (2.4) | .6640 | 2.7 (2.3) | 3.4 (2.5) | **.0053** |
| Active inflammation on MRI highly suggestive of sacroiliitis associated with SpA, ^d^ n (%) | 32 (47.8) | 29 (43.9) | .2073 | 75 (47.2) | 103 (55.7) | .3811 |

ASAS Assessment of Spondyloarthritis International Society; ASDAS-CRP, Axial Spondyloarthritis Disease Activity Score containing CRP; nr-axSpA, non-radiographic axial spondyloarthritis; NSAID, non-steroidal anti-inflammatory drug; SpA, spondyloarthritis; TNFi, tumor necrosis factor inhibitor.

^a^For the comparison of male versus female sex using Mann-Whitney and chi-square tests.

^b^SpA features included in the ASAS classification criteria for axSpA, excluding imaging.

^c^Based on patients with HLA-B27 assessed.

^d^As assessed by the investigator, the images could have been performed in the past.
