# Supplementary material for: Achieving inactive disease state in men and women with axial spondyloarthritis: results from a multi-country prospective observational study
Source: Rheumatology (Oxford). 2025 Aug 20;64(12):6337–44. doi: 10.1093/rheumatology/keaf447 (PMC12671858; doi:10.1093/rheumatology/keaf447)
Supplement: keaf447_Supplementary_Data [file keaf447_supplementary_data.zip › keaf447_Supplementary_Data/rhe-24-1010-File009.docx]

**Supplementary Table S3. Baseline characteristics stratified by TNFi use and sex in patients with r-axSpA**

| **Characteristic** | **TNFi use** | | | **No TNFi use** | | |
| --- | --- | --- | --- | --- | --- | --- |
|  | **Men**  **(n = 225)** | **Women**  **(n = 74)** | ***P* value^a^** | **Men**  **(n = 420)** | **Women**  **(n = 189)** | ***P* value^a^** |
| Age, y, mean (SD) | 35.4 (11.3) | 35.0 (10.5) | .8670 | 33.6 (11.1) | 35.6 (10.2) | **.0075** |
| Symptom duration, mo, mean (SD) | 64.0 (73.0) | 67.4 (81.2) | .8645 | 57.9 (96.0) | 66.9 (94.5) | .4888 |
| Time from diagnosis to baseline visit, mo, mean (SD) | 3.4 (3.4) | 4.3 (3.7) | .1402 | 2.8 (3.3) | 2.4 (3.0) | .3396 |
| Number of SpA features,^b^ mean (SD) | 4.2 (1.5) | 3.7 (1.3) | **.0160** | 3.9 (1.3) | 3.4 (1.3) | **.0003** |
| SpA features, n (%) |  |  |  |  |  |  |
| HLA-B27, positive^c^ | 139 (74.7) | 33 (53.2) | **.0015** | 260 (76.5) | 92 (56.8) | **<.0001** |
| Inflammatory back pain | 213 (94.7) | 72 (97.3) | .3528 | 404 (96.2) | 179 (94.7) | .4028 |
| Peripheral arthritis | 83 (36.9) | 32 (43.2) | .3297 | 133 (31.7) | 56 (29.6) | .6152 |
| Enthesitis, heel | 89 (39.6) | 24 (32.4) | .2730 | 130 (31.0) | 64 (33.9) | .4758 |
| Dactylitis | 14 (6.2) | 6 (8.1) | .5732 | 21 (5.0) | 6 (3.2) | .3113 |
| Uveitis | 34 (15.1) | 3 (4.1) | **.0122** | 42 (10.0) | 19 (10.1) | .9839 |
| Psoriasis | 13 (5.8) | 4 (5.4) | .9045 | 24 (5.7) | 7 (3.7) | .2963 |
| IBD | 6 (2.7) | 2 (2.7) | .9867 | 4 (1.0) | 4 (2.1) | .2431 |
| Good response to NSAIDs | 137 (60.9) | 46 (62.2) | .8454 | 250 (59.5) | 121 (64.0) | .2927 |
| Family history of SpA | 45 (20.0) | 12 (16.2) | .4722 | 79 (18.8) | 31 (16.4) | .4750 |
| Elevated CRP | 150 (66.7) | 36 (48.6) | **.0056** | 233 (55.5) | 63 (33.3) | **<.0001** |
| CRP, mg/L, mean (SD) | 21.8 (26.3) | 14.6 (21.9) | **.0041** | 17.3 (23.9) | 12.6 (21.2) | **.0001** |
| ASDAS-CRP, mean (SD) | 3.3 (1.2) | 3.2 (1.2) | .5762 | 2.9 (1.1) | 2.8 (1.1) | .8427 |
| BASDAI, mean (SD) | 4.9 (2.4) | 5.3 (2.5) | .1498 | 3.9 (2.1) | 4.5 (2.3) | **.0009** |
| BASFI, mean (SD) | 4.2 (2.6) | 4.4 (2.5) | .4711 | 2.8 (2.3) | 3.4 (2.5) | **.0082** |
| Active inflammation on MRI highly suggestive of sacroiliitis associated with SpA,^d^ n (%) | 24 (10.7) | 22 (29.7) | **<.0001** | 49 (11.7) | 45 (23.8) | **<.0001** |

ASAS Assessment of Spondyloarthritis International Society; ASDAS-CRP, Axial Spondyloarthritis Disease Activity Score containing CRP; r-axSpA, radiographic axial spondyloarthritis; NSAID, non-steroidal anti-inflammatory drug; SpA, spondyloarthritis; TNFi, tumor necrosis factor inhibitor.

^a^For the comparison of male versus female sex using Mann-Whitney and chi-square tests.

^b^SpA features included in the ASAS classification criteria for axSpA, excluding imaging.

^c^Based on patients with HLA-B27 assessed.

^d^As assessed by the investigator, the images could have been performed in the past.
